# Brevinin-1GHd: a novel *Hylarana guentheri* skin secretion-derived Brevinin-1 type peptide with antimicrobial and anticancer therapeutic potential

**DOI:** 10.1042/BSR20200019

**Published:** 2020-05-14

**Authors:** Yangyang Jiang, Yue Wu, Tao Wang, Xiaoling Chen, Mei Zhou, Chengbang Ma, Xinping Xi, Ying Zhang, Tianbao Chen, Chris Shaw, Lei Wang

**Affiliations:** 1 Natural Drug Discovery Group, School of Pharmacy, Queen’s University Belfast, Belfast BT9 7BL, Northern Ireland, U.K.; 2 Clinical Trial Center, Beijing Hospital, National Center of Gerontology, Institute of Geriatric Medicine, Chinese Academy of Medical Sciences, Beijing 10073, P.R. China

**Keywords:** anti-proliferation, antimicrobial peptides (AMPs), biofilms, Brevinin-1GHd, resistant strains

## Abstract

Host-defense antimicrobial peptides (AMPs) from amphibians are usually considered as one of the most promising next-generation antibiotics because of their excellent antimicrobial properties and low cytotoxicity. In the present study, one novel Brevinin-1 type peptide, Brevinin-1GHd, was isolated and characterized from the skin secretion of the frog, *Hylarana guentheri.* Brevinin-1GHd was found to possess a wide range of antimicrobial activity through penetrating the bacterial membrane within a short time while showing low hemolysis at bactericidal concentrations, even against the resistant strains. It also inhibited and eradicated biofilms that are thought to be closely related to the rise in resistance. Meanwhile, Brevinin-1GHd exhibited wide-spectrum anti-proliferation activity toward human cancer lines. Taken together, these results indicate that Brevinin-1GHd with its excellent antimicrobial and anticancer activities is a promising candidate for a novel antibiotic agent, and study of its structure–activity relationships also provided a rational template for further research and peptide analog design.

## Introduction

The growing problems of antibiotic-resistance have stressed the urgent demand for alternative and potent anti-infection agents [1,2]. As a fundamental constituent of innate immunity and defense systems, antimicrobial peptides (AMPs) from amphibian skin secretions have gradually become the focus of researchers. These skin-derived bioactive peptides were reported to have potent and broad-spectrum antimicrobial activity [3]. Compared with conventional antibiotics, AMPs are less likely to lead to resistance problems and can kill antibiotic-resistant bacteria within in a short time while showing low cytotoxicity to normal eukaryotic host cell lines, which makes them one of the most promising of alternative antibiotic agents [4].

Nowadays, more than 600 AMPs belonging to more than 30 families have been identified from amphibians [5]. Brevinins are a significant group of AMPs from the skin secretions of Rana frogs with potent antimicrobial activities and hemolytic actions. Generally, they have been divided into two subfamilies, Brevinin-1 and Brevinin-2, based on their primary structural characteristics. Brevinin-1 was first isolated from a skin extract of Rana *brevipoda porsa*, which is an Oriental pond frog [6]. They typically adopt an extended alpha-helix secondary structure and contain 24 amino acid residues and a heptapeptide ring known as a “Rana-Box” formed by a disulfide bond at the C-terminus [7]. Brevinin-1 family peptides exhibit a broad-spectrum antimicrobial activity including against some drug-resistant strains of pathogenic bacteria [8]. In addition to their excellent antibacterial activities, they also show strong anti-proliferation activity against a range of human tumor cells. However, their significant hemolytic activity would impede their further development as therapeutics [9].

In order to further study features of AMPs and find promising next-generation antibiotics, the work of our research group in isolating and characterizing novel bioactive peptides from frog skin secretions has continued. In this report, a novel AMP named Brevinin-1GHd was discovered in and isolated from the skin secretion of *Hylarana guentheri*. Through a ‘shotgun’ cloning strategy and structural confirmation by mass spectrum (MS/MS) fragmentation sequencing, the primary structure of Brevinin-1GHd was unequivocally established. A variety of biological and biophysical assays were subsequently applied to further evaluate the antimicrobial activity of Brevinin-1GHd on several common and drug-resistant micro-organisms and anti-proliferation activity against several common human cancer cells and cytotoxicity to horse red blood cells and normal human cells were also studied. The kinetics of bacterial killing and antibiofilm assays were also performed.

## Experimental section

### Specimen biodata and secretion acquisition

Specimens of the broad-folded Frog, *Hylarana guentheri* (*n* = 3), were obtained in the field in southern China. All frogs were adults of undetermined sex and secretion harvesting was performed in the field after which the frogs were released. Skin secretion was obtained by gentle transdermal electrical stimulation of the dorsal skin, as previously described [10]. Stimulated secretion was maintained at 4°C prior to being snap-frozen in liquid nitrogen, lyophilized and stored at −20°C prior to analyses. The procedure of secretion acquisition had been overseen by the Institutional Animal Care and Use Committee (IACUC) of Queen’s University Belfast, and approved on 1 March 2011. It was carried out under the U.K. animal (Scientific Procedures) Act 1986, Project license PPL 2694, which was issued by the Department of Health, Social Services and Public Safety, Northern Ireland.

### “Shotgun” cloning of cDNAs encoding novel peptide biosynthetic precursors

Lyophilized *Hylarana guentheri* skin secretion was dissolved (5 mg/ml) in cell Lysis/mRNA stabilization buffer (Dynal Biotech, U.K.). Polyadenylated mRNA was isolated by magnetic oligo-dT beads as described by the manufacturer (Dynal Biotech, U.K.) and subjected to 3′-RACE procedures to obtain full-length nucleic acid sequence encoding biosynthetic precursor using a SMART-RACE kit (Clontech, U.K.) essentially as described by the manufacturer. Briefly, the 3′-RACE reactions employed a Nested Universal Primer (supplied with the kit) and a degenerate sense primer (S1: 5′-GAWYYAYYHRAGCCYAAADATGTTCA-3′) that was designed to a highly conserved domain of the 5′-untranslated regions of previously characterized antimicrobial peptide precursor cDNAs from closely related Rana species. The PCR cycling procedure was as follows—initial denaturation step: 60 s at 94°C; 35 cycles: denaturation 30 s at 94°C, primer annealing for 30 s at 61°C; extension for 180 s at 72°C. PCR products were gel-purified, cloned using a pGEM-T vector system (Promega Corporation) and sequenced using an ABI 3100 automated capillary sequencer.

### Analyses of Brevinin-1GHd primary and secondary structure

Reverse-phase HPLC (RP-HPLC) and mass spectrometry were used to elucidate the primary structure of Brevinin-1GHd. A further 5 mg of lyophilized *Hylarana guentheri* skin secretion were analyzed as in a previous study [11]. An analytical RP-HPLC column (Phenomenex C-5, 0.46 cm × 25 cm) and Cecil CE4200 Adept (Cambridge, U.K.) gradient RP-HPLC system (from 0.05/99.95 (v/v) trifluoroacetic acid (TFA)/water to 0.05/19.95/80.00 (v/v/v) TFA/water/acetonitrile in 80 min) were employed to isolate peptides from skin secretion. Thereafter, the primary structure of the novel peptide was established by a matrix-assisted laser desorption ionization, time-of-flight mass spectrometry (MALDI-TOF MS) (Voyager DE, Perspective Biosystems, Foster City, CA, U.S.A.) and an LCQ-Fleet electrospray ion-trap mass spectrometer (Thermo Fisher Scientific, San Jose, CA, U.S.A.).

Bioinformatics techniques were used to analyze the acquired sequence. An online tool, NCBI-BLAST (https://blast.ncbi.nlm.nih.gov/Blast.cgi), was used to compare this sequence with all the sequences that are recorded in GenBank, and then generated related peptide sequences based on identities ranging from high to low. The helical structure and structural parameters were analyzed by HeliQuest (http://heliquest.ipmc.cnrs.fr/) and I-TASSER online server.

The secondary structure of Brevinin-1GHd was determined with the JASCO J-815 circular dichroism (CD) spectrometer (Jasco, Essex, U.K.). About 50 μM of peptide solutions were prepared in precision quartz cell (Hellma Analytics, Essex, U.K.) with 10 mM ammonium acetate (NH_4_AC) and 50% trifluoroehanol (TFE) in 10 mM NH_4_AC buffer, respectively. The sample was analyzed at 20°C with scan range of 190–250 nm, scanning speed of 100 nm/min, 1 nm bandwidth and 0.5 nm data pitch.

### Peptide synthesis and purification

The peptide was synthesized by a solid phase peptide synthesis method using a synthesizer (Protein Technologies, U.S.A.) along with standard Fmoc-chemistry. The primary peptide product was cleaved from the resin and deprotected for at least 8 h, precipitated in ether over 3 days, and then washed three times with ether and dried for more than 24 h. After this, the peptide was re-dissolved and rapidly frozen in liquid nitrogen and lyophilized in a freeze dryer. After freeze drying, the peptide was purified by RP-HPLC.

### Minimal inhibitory concentration (MIC) and minimal bactericidal concentration (MBC) assays

MIC assays of synthetic peptide were determined using a standard Gram-positive bacterium, *S. aureus* (NCTC 10788), a typical Gram-negative bacterium, *E. coli* (NCTC 10418), a classic yeast, *C. albicans* (NCPE 1467), a drug resistant Gram-negative bacterium, *P. aeruginosa* (ATCC 27853) and a methicillin-resistant *S. aureus*, (MRSA) (NCTC 12493). Brevinin-1GHd was initially dissolved in DMSO to a concentration of 5.12 × 10^4^ µM, then 2-fold diluted until the concentration of 100 µM. All micro-organisms were first incubated in Mueller-Hinton broth (MHB) (Sigma-Aldrich, St. Louis, MO, U.S.A.) for 16–18 h. Then, 500 µl of bacterial cultures were transferred to 20 ml of MHB and cultured in a shaking incubator at 37°C until reaching log phase. The bacterial cultures were then diluted with MHB to 10^6^ colony forming units (CFU) /ml and added into wells of 96-well plates with Brevinin-1GHd in a range of final concentration from 512 to 1 µM. After incubating at 37°C overnight, the growth of bacteria and yeast were measured by optical density (OD) values at 550 nm by an ELISA plate reader (Biotech, Minneapolis, MN, U.S.A.). For wells without obvious growth of micro-organisms, 20 µl of solutions were set on Mueller Hinton agar (MHA) plates. MHA plates were then placed in an incubator at 37°C overnight, and the lowest concentration without micro-organism colonies were the MBC.

### Kinetic time-killing assay

To evaluate the antimicrobial properties, the kill-time curve assay was performed against the Gram-negative bacterium, *E. coli*, the drug resistant Gram-negative bacterium, *P. aeruginosa*, the Gram-positive bacterium, *S. aureus* and MRSA. Cell cultures containing various concentrations (0.5 ×, 1 ×, 2 × MIC) of peptide and 32 μM of ampicillin were diluted with phosphate-buffered saline (PBS) and spread over MHA plates after incubation for 0, 5, 10, 20, 30, 60 and 120 min. The bacterial colonies were counted after overnight incubation.

### Minimum biofilm inhibitory concentration (MBIC) and minimum biofilm eradication concentration (MBEC) assays

MBIC and MBEC were performed as previously described [11]. In MBIC assay, bacteria culture (5 × 10^5^ CFU/ml) was incubated with different concentrations of Brevinin-1GHd (ranging from 521 to 1 μM) at 37°C for 24 h. In MBEC assay, 100 μl of bacteria medium was seeded to a 96-wells plate and incubated at 37°C for 48 h until the appearance of mature biofilm. Then, the plate was washed with sterile PBS twice and treated with different concentrations of Brevinin-1GHd at 37°C for 24 h. The plate was then washed with PBS for twice and stained with Crystal Violet solution (Sigma-Aldrich, U.K.), and further dissolved with 30% of acetic acid (Sigma-Aldrich, U.K.). The absorbance was measured at 595 nm with a Synergy HT plate reader (Biotech, Minneapolis, MN, U.S.A.).

### SYTOX green assay

The SYTOX green assay was performed as previously reported to analyze the effect of peptide on membrane integrity [11]. Briefly, bacterial strains were incubated to the exponential phase and collected after centrifuging at 1000 × ***g*** for 10 min. Then, the bacterial pellet was washed with 5% TSB (dissolved in 0.85% NaCl solution) twice and resuspended at a density of 10^8^ CFU/ml. Peptide at concentrations of 1× MIC, 2 × MIC and 4 × MIC were incubated with bacterial suspension at 37°C for 2 h and then mixed with SYTOX Green nucleic acid stain (Thermo Fisher Scientific, U.S.A.) at 37°C for 10 min on the shaking incubator. The changes of fluorescence were monitored by use of a Synergy HT plate reader at excitation/emission wavelengths of 485/528 nm.

### Hemolysis and cytotoxicity assays

The hemolysis assay used horse erythrocytes (2% suspension) (TCS Biosciences Ltd., Buckingham, U.K.) as reported previously [11]. Serial concentrations of peptide were incubated with horse blood cell suspension at 37°C for 2 h. 1% Triton X-100 was used as positive control to calculate hemolysis of test peptide and PBS was served as the negative control. After that, the mixtures were centrifuged at 1000 × ***g*** for 5 min and lysis of red cells was determined by measuring of the OD values at 550 nm.

Furthermore, the cytotoxicity of Brevinin-1GHd against human microvascular endothelial cells (HMEC-1) (ATCC-CRL-3243) and human keratinocytes (HaCaT) (ATCC-PCS-200-011) were evaluated as reported [12]. HMEC-1 cell line (5 × 10^3^ cells/ml) was seeded into a 96-well plate with MCDB131 medium (Life technologies, Carlsbad, CA) supplemented with 10% (v/v) fetal bovine serum (FBS), 1% (v/v) penicillin-streptomycin, 10 ng/ml epidermal growth factor, 10 mM L-glutamine and 1 µg/ml hydrocortisone for 24 h. HaCaT (5 × 10^3^ cells/ml) was seeded into a 96-well plate with DMEM medium (Life technologies, U.K.) supplemented with 10% (v/v) FBS, 1% (v/v) penicillin–streptomycin. After that, cells were treated with corresponding serum-free culture medium for 4 h. Cells were then treated with Brevinin-1GHd in concentrations ranging from 10^−9^ to 10^−4^ M. After 24 h of treatment, 10 µl of MTT reagent (5 mg/ml) was added to each sample and cells were incubated at 37°C. After exposure for 4 h, all liquid were removed and 100 µl of DMSO were added and the absorbance of each sample at 570 nm was determined using an ELISA plate reader.

### Human cancer cell MTT assay

The anticancer activity of peptide on four kinds of cancer cell lines: H157 (ATCC-CRL-5802), U251MG (ECACC-09063001), MDA-MB-435s (ATCC-HTB-129) and PC3 (ATCC-CRL-1435) were measured by the MTT assay. Cell lines were seeded into 96-well plates at a concentration of 5 × 10^3^ cells per well in the corresponding culture medium with serum for 24 h. The next day, the culture medium was replaced with 100 μl of serum-free medium. After 4 h, Brevinin-1GHd solutions, in concentrations ranging from 0.1 nM to 0.1 mM in serum-free medium, were added to replace the culture medium. After 24 h of treatment, 10 µl of MTT reagent (5 mg/ml) were added to each sample. The cells were incubated for 4 h and mixtures in each well were then removed and replaced with 100 µl of DMSO. Finally, the absorbance of each sample at 570 nm was determined using an ELISA plate reader.

### Statistical analysis

Statistical analyses of all bioactivity assays were performed using Prism 6 (GraphPad Software, U.S.A.). The statistical significance of difference was analyzed by a one-way ANOVA. Data points represent the average of three independent experiments with error bars presenting the SEM.

## Results

### “Shotgun” cloning of Brevinin-1GHd from the skin secretion of *Hylarana guentheri*

**Figure 1:**
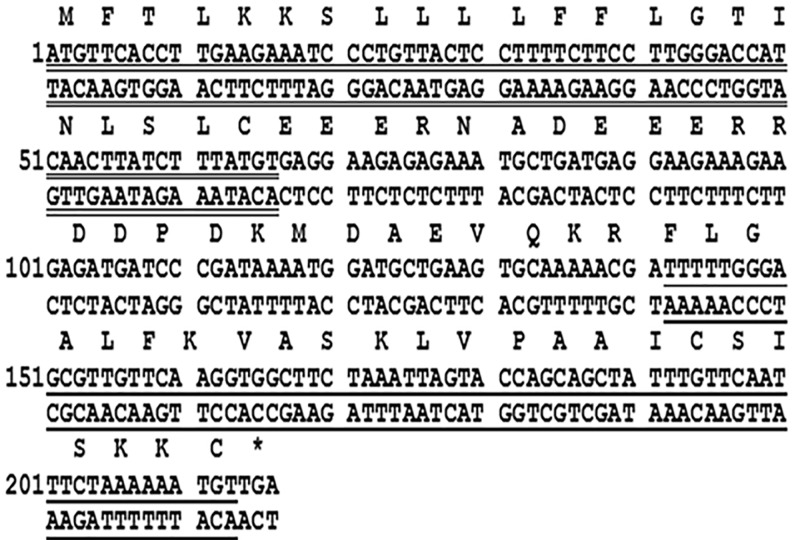
Nucleotide and translated open reading frame amino acid sequence of cDNA encoding the Brevinin-1GHd peptide precursor cloned from the skin secretion library of *Hylarana guentheri* The putative signal peptide (double-underlined), mature peptide (single-underlined) and stop codon (asterisk), are indicated.

A biosynthetic precursor encoding Brevinin-1GHd cDNA was consistently cloned from the skin secretion cDNA library of *Hylarana guentheri*. The open reading frame consisted of 71 amino acid residues. The N-terminal 22 amino acid residues encoded a putative signal peptide, followed by an acidic amino acid residue-rich spacer domain that terminated in a typical -Lys-Arg- (-KR-) propeptide convertase processing site, followed immediately by the mature AMP at the C-terminal end (Figure 1). The average molecular mass of the putative mature peptide from the cloned precursor was calculated as 2495.14 Da. The Brevinin-1GHd biosynthetic precursor-encoding cDNA has been deposited in the NCBI Database (accession code; MN817129).

### Brevinin-1GHd primary and secondary structure

**Figure 2:**
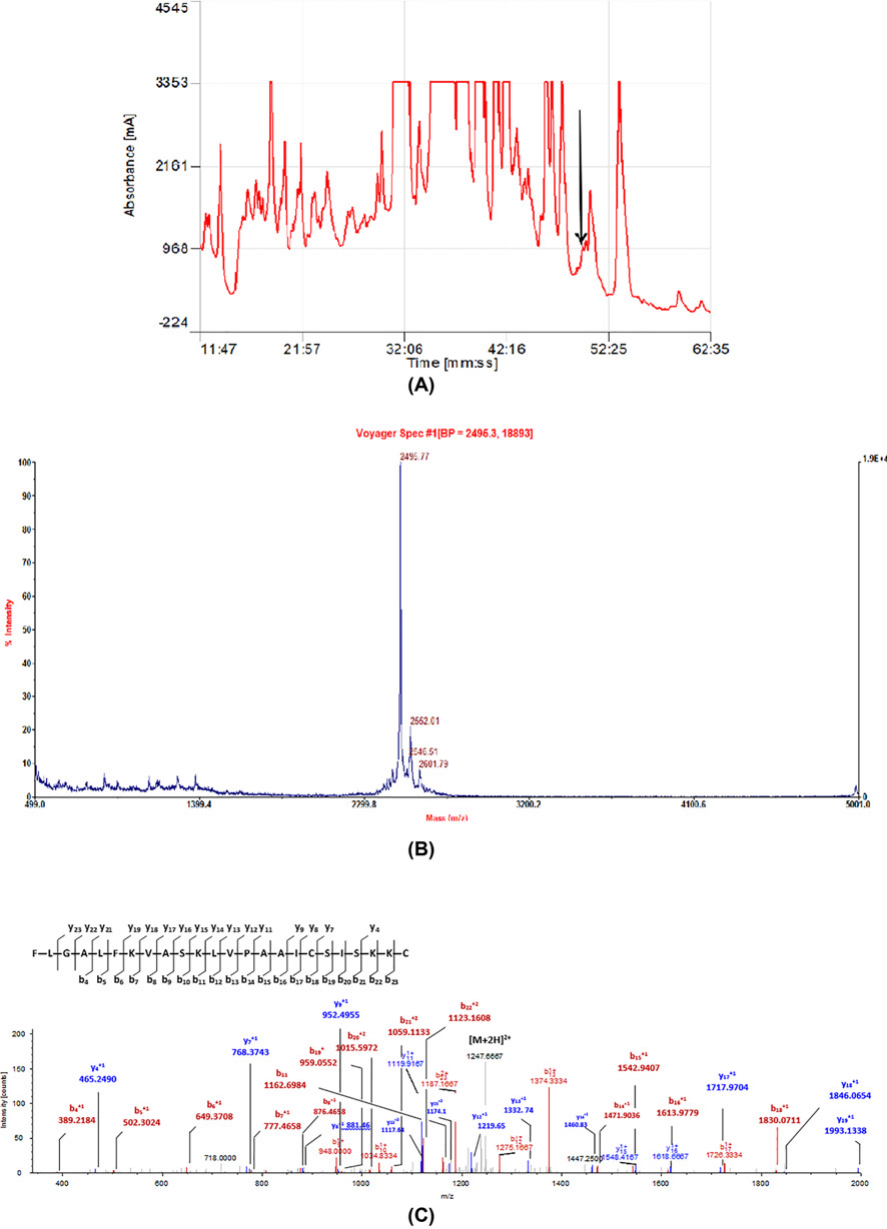
Chromatogram of *Hylarana guentheri* skin secretion and corresponding mass spectra of Brevinin-1GHd (**A**) Region of RP-HPLC chromatogram of *Hylarana guentheri* skin secretion with the elution position/retention time of Brevinin-1GHd indicated by an arrow. Absorbance wavelength was set at 214 nm. (**B**) MALDI-TOF mass spectrum of the HPLC fraction at 50 min in panel (A) corresponding to Brevinin-1GHd. (**C**) Thermoquest LCQ™ fragment scan spectrum derived from ions corresponding to Brevinin-1GHd. Observed ions following MS/MS fragmentation are indicated in color.

The putative mature peptide, Brevinin-1GHd, was identified by RP-HPLC through analyzing the lyophilized crude skin secretion of *Hylarana guentheri*. All collected fractions were then analyzed by MALDI-TOF MS and a single fraction containing a peptide corresponding to the theoretical molecular mass was identified (Figure 2A,B). The primary structure of Brevinin-1GHd was further analyzed by MS/MS fragmentation sequencing (Figure 2C).

**Figure 3:**
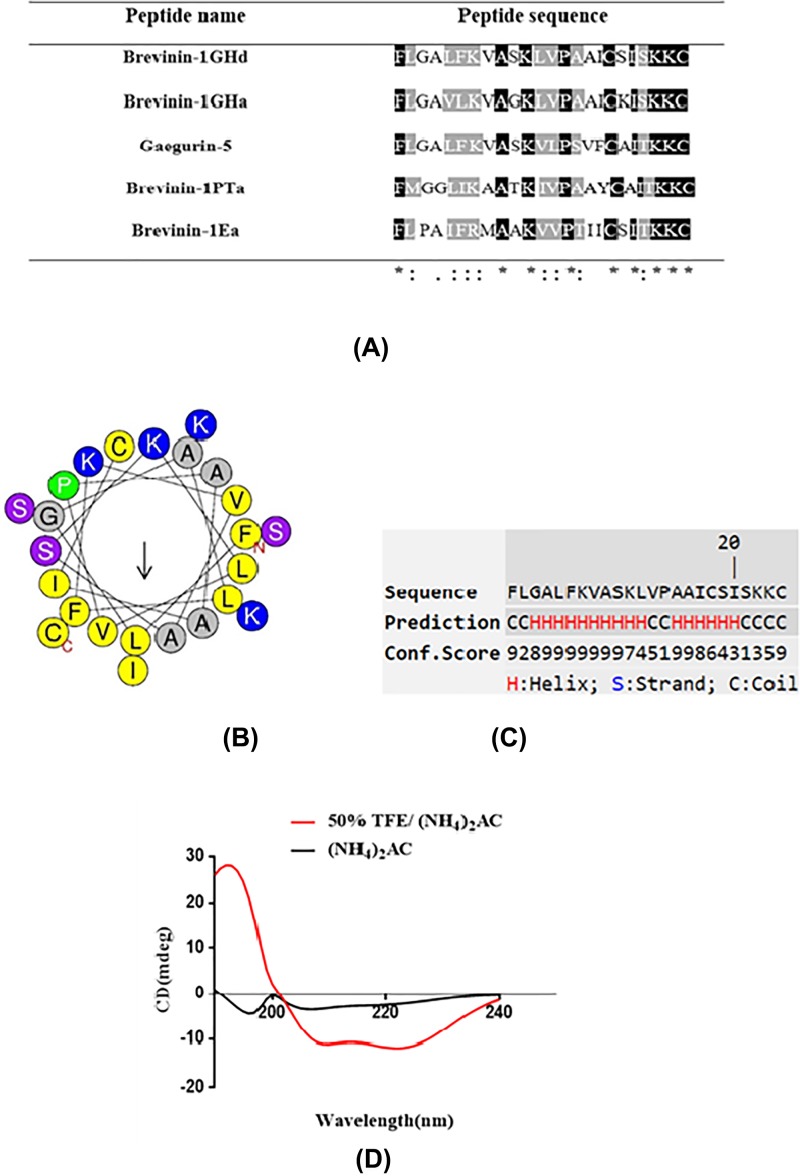
Bioinformatics and secondary structure analyses of Brevinin-1GHd (**A**) Alignment of mature peptide Brevinin-1GHd with similar AMPs of the Brevinin-1 subfamily found in the NCBI database. Identical amino acids residues are indicated by asterisks. (**B**) Helical wheel projection of Brevinin-1GHd with an arrow indicated the hydrophobic face. (**C**) Predicted secondary structure with a dominated alpha-helix structure. (**D**) Secondary structures of Brevinin-1GHd. The CD spectra of peptides were measured in an aqueous environment (10 mM NH_4_AC buffer) and a membrane-mimetic environment (50% TFE in 10 mM NH_4_AC), respectively.

According to the bioinformatics analysis using the NCBI-BLAST program, Brevinin-1GHd shared considerable sequence similarity with Brevinin-1 subfamily peptides (Figure 3A). Brevinin-1GHd was the second Brevinin-1 type peptide found in *Hylarana guentheri* skin secretion after Brevinin-1GHa. The alignment result showed that Brevinin-1GHd shared 83% sequence identity with Brevinin-1GHa.

To further study putative secondary structure and structural parameters, Heliquest and I-TASSER were used in analysis (Figure 3B,C). The results demonstrated that Brevinin-1GHd adopted an alpha-helix secondary structure (Figure 3D) with a hydrophobic face consisting of A, A, L, I, V (Table 1).

**Table 1:** Structural parameters of Brevinin-1GHd

_1_FLGALFKVASKLVPAAICSISKKC_24_		
Physico-chemical properties	Polar residues + GLY	None-polar residues
**Hydrophobicity <H>**	**Polar residues + GLY (n / %)**	**Nonpolar residues (n / %)**
0.653	8/33	16/66.7
**Hydrophobic moment <µH>**	**Uncharged residues + GLY**	**Aromatic residues**
0.364	SER 3, GLY 1	PHE 2,
**Net charge z**	**Charged residues**	**Special residues**
4	LYS 4,	CYS 2, PRO 1
**Hydrophobic face:** A A L I V		

The results of CD spectroscopy revealed that Brevinin-1GHd adopt a random coil structure in an aqueous environment, while forming an alpha-helix structure in a membrane-mimetic environment.

### MIC and MBC

Brevinin-1GHd was found to be active against the yeast, *C. albicans*, with an MIC of 4 µM and was even more potent against the Gram-positive bacteria, *S. aureus* and MRSA, with MICs of 2 and 4 µM, respectively (Table 2). However, this peptide was less potent against the Gram-negative bacteria, *E. coli* and *P. aeruginosa*, with an MICs of 8 and 32 µM. Notably, the antimicrobial activities of Brevinin-1GHd against *P. aeruginosa* were comparable to *Ampicillin*.

**Table 2:** MICs and MBCs of Brevinin-1GHd, melittin, *Ampicillin*

Treatments	MIC/MBC (µM)				
	*S. aureus*	MRSA	*E. coli*	*P. aeruginosa*	*C. albicans*
Brevinin-1GHd	2/4	4/4	8/8	32/32	4/8
Melittin	2/2	2/4	2/2	32/32	4/4
*Ampicillin*	0.3/0.3	-	4.8/4.8	36.6/36.6	146/-

### Time-killing assay for Brevinin-1GHd against *E. coli, S. aureus, P. aeruginosa* and MRSA

**Figure 4:**
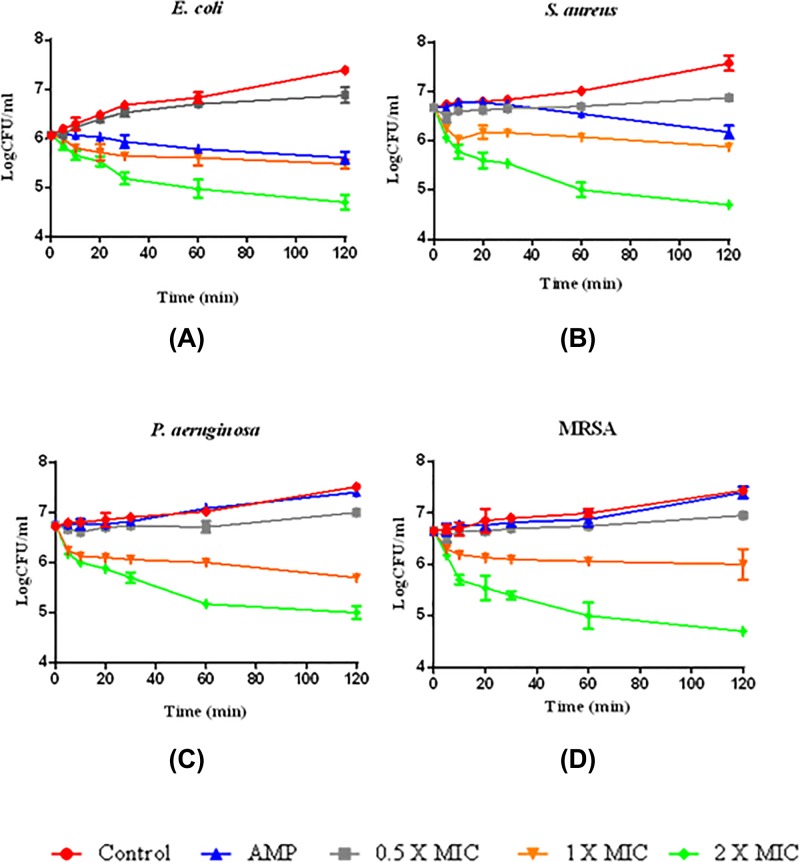
Time-kill curves of Brevinin-1GHd Responses of 2-fold MICs (green), 1-fold MICs and 0.5-fold MICs (grey) of peptide over 120 min were plotted against four different strains (**A**) *E. coli*, (**B**) *S. aureus*, (**C**) *P. aeruginosa* and (**D**) MRSA. The *X*-axis is time in min, the *Y*-axis is CFU/ml. AMP (blue) and control (red) represent *Ampicillin* (32 µM) and control group (no drug), respectively.

The time-killing assay is widely used for *in vitro* investigations of novel AMPs. In the present work, two common bacteria, *E. coli* (Figure 4A) and *S. aureus* (Figure 4B), and two drug-resistant bacteria, *P. aeruginosa* (Figure 4C) and MRSA (Figure 4D), were subjected to kinetic study. The novel peptide displayed rapid bactericidal activity against all test strains, showing a higher efficiency compared to *Ampicillin* after 1 h exposure (Figure 4).

### Anti-biofilm activity of Brevinin-1GHd

Brevinin-1GHd was found to inhibit biofilm formation of *S. aureus* and MRSA at concentrations of 2 and 4 µM, respectively. Brevinin-1GHd also eradicated established biofilms at concentrations of 2 and 16 µM.

### Membrane permeability assay

**Figure 5:**
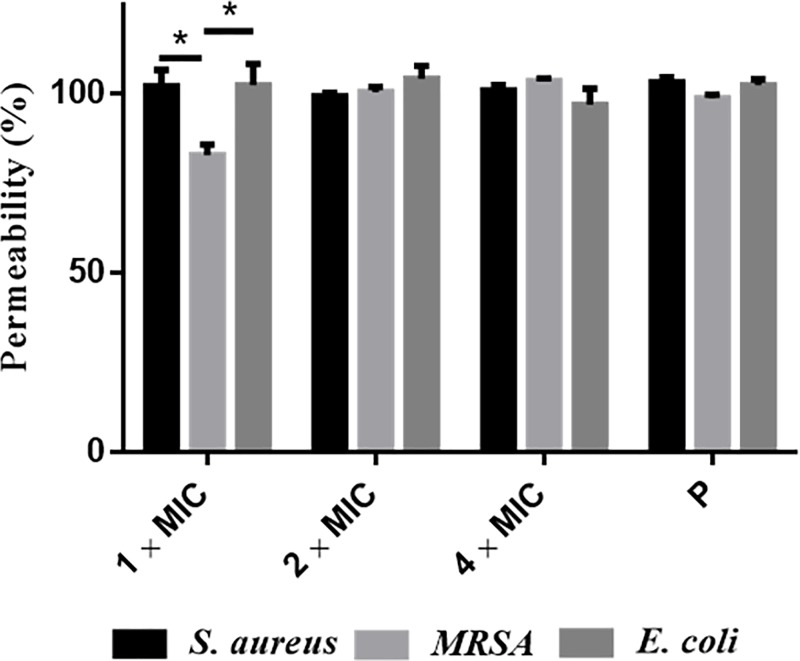
Cell permeability of *S. aureus*, MRSA and *E. coli* treated with Brevinin-1GHd at 1-fold, 2-fold and 4-fold of MIC The cells permeabilized by 70% isopropanol were used as the positive control (P). *P < 0.05 versus MRSA incubated with 1-fold MIC of Brevinin-1GHd.

Brevinin-1GHd induced 100% permeability of cell membranes at 2 and 4-fold MIC. At 1-fold MIC, Brevinin-1GHd showed slightly higher membrane permeability on *S. aureus* (100%) and *E. coli* (100%) than on MRSA (83%) (Figure 5).

### Hemolytic and cytotoxic activity

**Figure 6:**
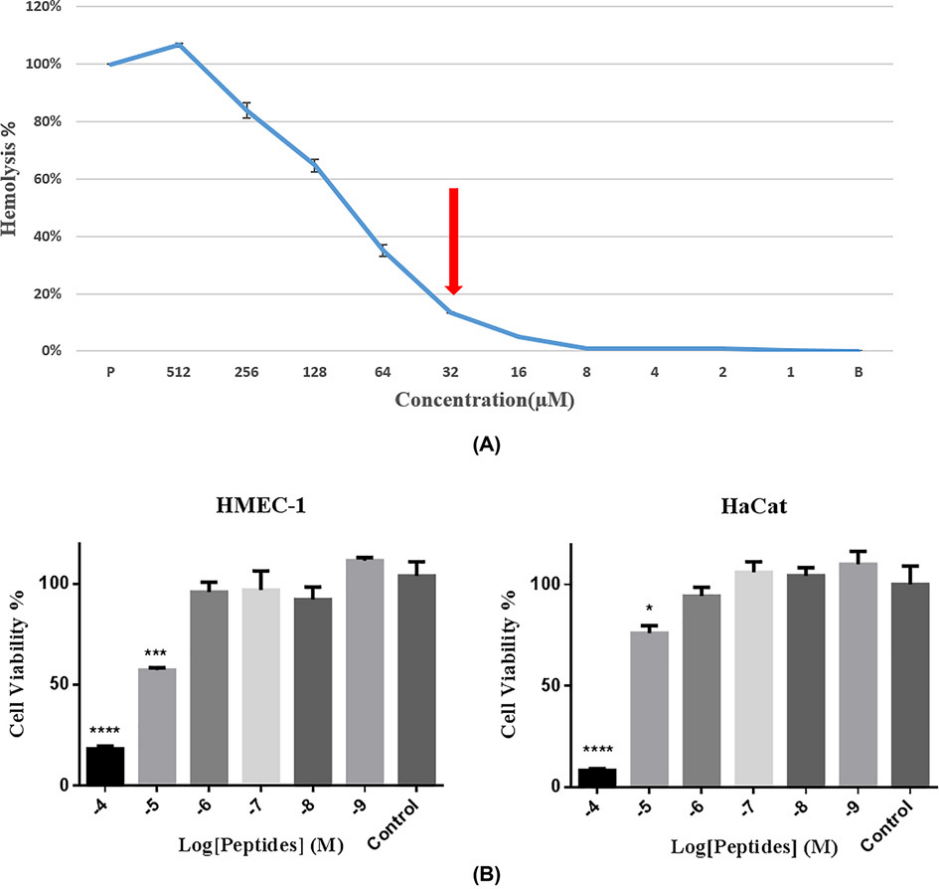
Hemolytic and cytotoxic activity of Brevinin-1GHd (**A**) Hemolytic activity of Brevinin-1GHd. The highest MIC/MBC value of the tested bacteria *P. aeruginosa* (32 µM) is indicated by the red arrow. P and B represent positive control (1% Triton X-100) and negative control (PBS), respectively. (**B**) Dose-dependent anti-proliferative effects of Brevinin-1GHd against HMEC-1 cells (left) and HaCaT cells (right); *P < 0.05, ***P < 0.001, ****P < 0.0001 in comparison with control group (no drug).

Brevinin-1GHd displayed 13% hemolysis at its highest MIC/MBC value, indicating Brevinin-1GHd exhibited low hemolytic activity on horse red blood cells (Figure 6A). However, when the concentration increased above 64 μM, the hemolytic activity of Brevinin-1GHd also gradually increased. In addition, Brevinin-1GHd showed significant cytotoxicity toward human HMEC-1 and HaCaT cells at concentrations of 10^−5^ and 10^−4^ M, respectively (Figure 6B).

### Anti-cancer activity

**Figure 7:**
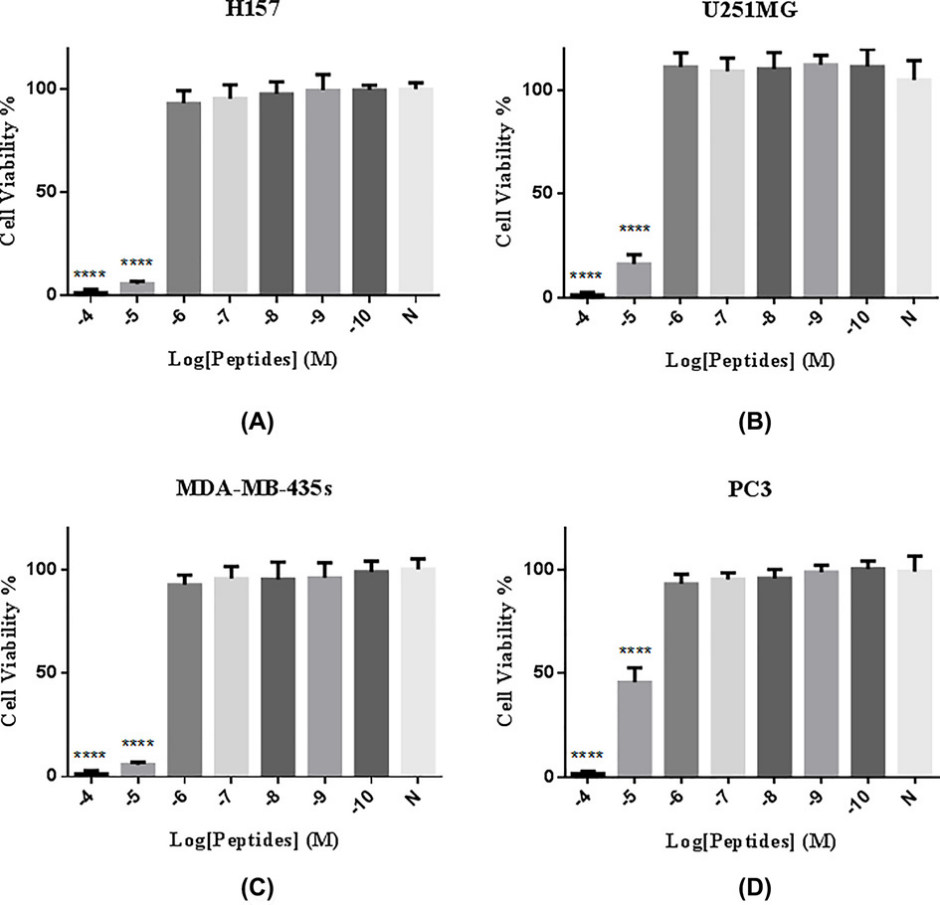
Anti-proliferation activity of Brevinin-1GHd on human cancer cell lines (**A**) H157, (**B**) U251MG, (**C**) MDA-MB-435s and (**D**) PC3. Each cell line was treated with Brevinin-1GHd in a range of concentrations from 10^−10^ to 10^−4^ M. N means negative control group (no drug). ****P < 0.0001 in comparison with negative control group.

The ability of Brevinin-1GHd to inhibit the proliferation of different human cancer cell lines and an epithelial cell line, at concentrations between 10^−4^ and 10^−10^ M, was assessed using the MTT assay (Figure 7). Brevinin-1GHd exhibited obvious anti-proliferation activity on H157 cells (non-small cell lung cancer), U251MG cells (human neuronal glioblastoma (astrocytoma)), MDA-MB-435s cells (melanoma), and PC3 cells (human prostate carcinoma), with IC_50_ values of 2.987, 7.985, 1.197 and 9.854 µM, respectively (Table 3).

**Table 3:** IC_50_ values of Brevinin-1GHd calculated from the normalized curves in Figures 6B and 7

Cells	IC_50_ (µM)
**HMEC-1**	15.62
**HaCaT**	29.69
**H157**	2.987
**U251MG**	7.985
**MDA-MB-435s**	1.197
**PC3**	9.854

## Discussion

The skins of amphibians play an essential role in their survival and their ability to thrive in complex environments. Gene-encoding AMPs are one of the most important components of amphibian innate immunity and defense system against infection by micro-organisms. In the present paper, one novel AMP was isolated and characterized from skin secretion of *Hylarana guentheri*. As the NCBI-BLAST alignment suggested, this novel peptide shared similar properties with most peptides in the Brevinin-1 subfamily. Among these, Brevinin-1GHd is 83% identical with Brevinin-1GHa and 71% identical with Gaegurin-5 [13,14].

### Antimicrobial activity

As with most peptides of the Brevinin-1 family, Brevinin-1GHd adopted an alpha-helical structure and exhibited a wide range of antimicrobial activity, even against resistant strains such as MRSA and *P. aeruginosa*. Among these, Brevinin-1GHd presented a higher potency against the typical Gram-positive bacterium, *S. aureus*, when compared with the classical Gram-negative bacterium, *E. coli*, and this phenomenon is quite commonly observed for many other peptides. The fact that Brevinin-1GHd showed stronger activity against *S. aureus* than against *E. coli*, could be explained by the differences in structures of their cell envelopes. Gram-positive bacteria have a thicker peptidoglycan layer consisting of anionic teichoic acid. In contrast, in Gram-negative bacteria, the peptidoglycan layer is thinner and less cross-linked. Also, Gram-negative bacteria have a thick lipopolysaccharide (LPS) layer outside the peptidoglycan layer [15]. When the AMPs attack Gram-positive bacteria, they only need to diffuse across the layer of peptidoglycan first and then disrupt the cytoplasmic membrane. However, in the case of Gram-negative bacteria, AMPs must permeabilize a thick LPS layer before they reach and disrupt the inner cytoplasmic membrane [16]. The result of time-killing kinetic assays and SYTOX green assays, revealed that Brevinin-1GHd killed the bacteria within a short time mainly through permeating and disrupting their membranes.

### Anticancer activity

Brevinin-1GHd showed cytotoxicity on normal human cells HMEC-1 and HaCaT with IC_50_ values of 15.62 and 29.69 µM, respectively; however, IC_50_ values of Brevinin-1GHd toward test cancer cells were relatively lower (Table 3), demonstrating, at working concentrations, Brevinin-1GHd could inhibit the growth of test cancer cells without causing huge damage on normal cells. Therefore, the further development of Brevinin-1GHd in anticancer study will be still promising. According to previous studies, Brevinin-1 type peptides were reported to inhibit the growth of a wide range of cancer cells [9]. However, the exact mechanism of AMP action is still not clear yet [17]. It is generally accepted that cancer cell membranes, unlike those of normal cells, display a negatively charged surface. The cationic AMPs could thus combine with the cancer cell membrane through electrostatic interaction [17]. When the AMPs accumulate on the bacterial outer membrane to reach a certain concentration, AMPs can penetrate the membrane, causing leakage of the cellular contents leading to cell death. This mechanism may explain the action of Brevinin-1GHd since Brevinin-1GHd contained four net charges, which may allow the peptide to interact with the anionic membrane.

### Hemolysis and cytotoxicity

Although Brevinin-1GHd showed considerable hemolysis at high concentration, it did not possess significant hemolytic activity at the lowest MIC concentration. Hemolytic activity is a common characteristic for most amphibian-derived AMPs, and this factor can impede the further therapeutic development of most AMPs [18]. However, through rational design and structural modification, this problem can be solved to some extent and the application of these AMPs could still be promising. Meanwhile, although Brevinin-1GHd showed significant cytotoxicity on normal human cells at 10^−4^M, this side-effect was greatly reduced at the concentration of 10^−5^M under which the growth of cancer cells was still inhibited. Therefore, the further development of this peptide is still promising.

**Figure 8:**
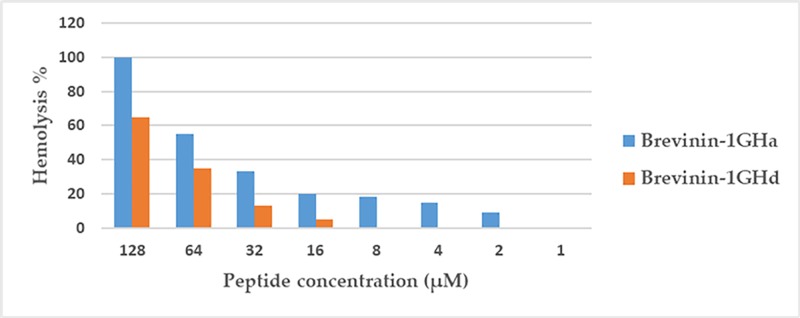
Comparison of hemolytic activity between Brevinin-1GHa and Brevinin-1GHd

In our previous study, one novel Brevinin-1 type peptide, Brevinin-1GHa, was first found in *Hylarana guentheri* and two analogs, Brevinin-1GHb and Brevinin-1GHc, were designed to further study the relationships between structure and activity. The study of Brevinin-1GHa and its two analogs revealed that the deletion or removal of the “Rana-Box” decreased its antimicrobial activity while reducing hemolytic activity [13]. Through comparing differences of structure and activity between Brevinin-1GHa and its analogs, the hypothesis was established that the hemolytic activity was determined both by the hydrophobicity and hydrophobic face since the lack and decrease in hydrophobicity of Brevinin-1GHb and Brevinin-1GHc showed lower hemolytic activity than Brevinin-1GHa [13]. Interestingly, although Brevinin-1GHd had the same hydrophobic face, higher hydrophobicity and similar length of alpha-helix structure as Brevinin-1GHa, its hemolytic activity was slightly lower than Brevinin-1GHa (Figure 8). By further comparing their structural parameters (Table 4), we decided to supplement the hypothesis that besides the effects of hydrophobicity and hydrophobic face on peptide hemolytic performance, the role of net charge also needs to be considered. Since fungal membranes are similar with those of other eukaryotic cells, the trend of anti-fungal activity may be similar with that of hemolytic activity [19]. Compared with Brevinin-1GHa, the net charge of Brevinin-1GHd was slightly lower, which may explain the different antimicrobial activity against *C. albicans*. Normally, the hemolysis of eukaryotic cells requires peptide to insert into the cell membrane and form a channel-like structure as in the “barrel-stave” model. The higher net charge the peptides contain, the easier they bind to the cell membrane [20]. Therefore, although Brevinin-1GHd had higher hydrophobicity, similar length of alpha-helix and same hydrophobic face, it showed relative lower cytotoxicity toward red blood cells than Brevinin-1GHa.

**Table 4:** Structural parameters and MICs of Brevinin-1GHd and relevant AMPs homologs against specified micro-organisms

Peptide name	Net charge	Hydrophobicity <H>	Antimicrobial activity (µM)			
			*S. aureus*	*E. coli*	*C. albicans*	*MRSA*
Brevinin-1GHa	+5	0.592	2	4	2	4
Brevinin-1GHb	+2	0.725	512	>512	>512	>512
Brevinin-1GHc	+5	0.592	32	32	128	128
Brevinin-1GHd	+4	0.653	2	8	4	4

### The role of the “Rana-Box”

The “Rana-Box” is a unique structure and appears in many AMPs originating from frogs. This contains an intermolecular disulfide bridge at the C-terminus of the peptide and seven to nine amino acid residues [21]. According to previous studies, the roles of the “Rana-Box” could be briefly summarized in several points: (1) The “Rana-Box” stabilizes the alpha-helix structure, complementing a stable activity; (2) The cyclic structure provides a stable structure for the peptide to resist the hydrolysis of proteases such as carboxypeptidase; (3) The “Rana-Box” favors peptide to induce membrane conductance in planar lipid layers and the efflux of K^+^ from bacteria; (4) For some peptides, the “Rana-Box” provides the source of net positive charge [22].

In the past decades, a large number of studies have been carried on the “Rana-Box”. Some studies emphasized the importance of this structure since elimination of the C-terminal cysteine residue impeded antimicrobial performance [14]. Other studies, however, dismissed its role as replacing cysteine residues with serine, deletion of disulfide bond or resetting the position of the “Rana-Box” produced no effect on peptide antimicrobial activity, even helping to reduce hemolysis [22–25]. In a previous study, we also characterized and designed Brevinin-1GHa and its two analogs to further study the relationship of the “Rana-Box” structure and its effect on antimicrobial activity. However, neither truncating the C-terminal cyclic structure nor removing the “Rana-Box” to the middle part of the peptide, greatly decreased antimicrobial activity [13]. The actual role of the “Rana-Box” in antimicrobial performance is still ambiguous and more factors need to be considered. In fact, no matter what changes in this cyclic structure were made, these would always come with some variations on structural parameters or secondary structure. For example, truncating the “Rana-Box” greatly increased the helicity of Nigrocin-HL analog, which may explain why the antimicrobial activity of Nigrocin-HLD was better [12]. However, the elimination of cyclic structure made the putative length of the alpha-helix of Brevinin-1GHb shorter than Brevinin-1GHa, which was in accordance with the decrease of antimicrobial activity. Also, the motif of the cyclic structure was quite different between different families, even for the peptides in the same family. The variations in residues may lead to differences in structural parameters, which may further influence the working model of the peptide. Therefore, analyzing the relationship of the “Rana-Box” structure and activity needs to be carried out on the case-by-case basis.

**Figure 9:**
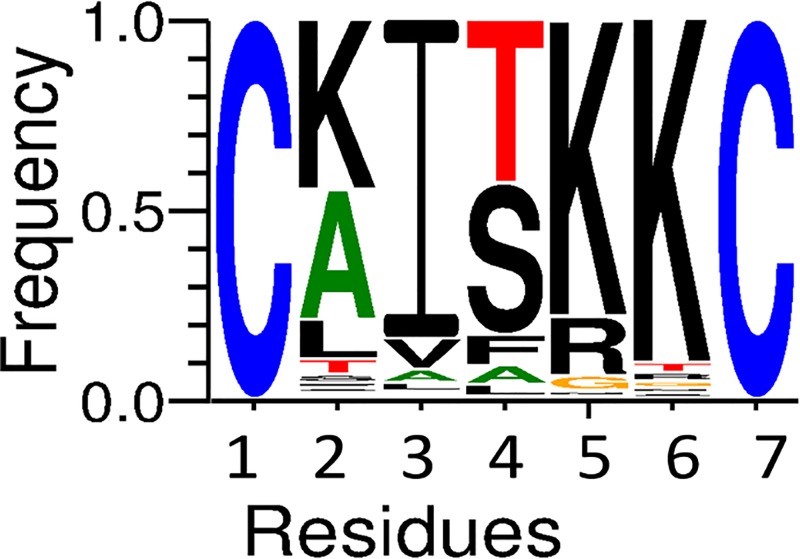
Sequence diversity in the “Rana-Box” loop of heptapeptide-ring containing Brevinin-1 family peptides This heptapeptide loop sequences were obtained from previous reported Brevinin-1 family peptides (those containing heptapeptide rings) alignment illustrated with a sequence logo graphic. The *X*-axis demonstrates the diversity of residues at each position and the *Y*-axis shows the relative frequency.

In order to further characterize this cyclic structure in the Brevinin-1 family, we collected and aligned all Brevinin-1 peptides from the UNIPORT database (https://www.uniprot.org/). In the Brevinin-1 family, most peptides contained seven amino acids within their “Rana-Box”. For each heptapeptide-ring containing Brevinin-1 family peptide, the sequence of each loop segment was extracted and submitted to WebLogo (URL: http://weblogo.threeplusone.com) to generate a logo graphic (Figure 9).

Residue preferences identified in Figure 9 revealed the molecular diversity and residue specificity. The most prevalent residue at each position was also the most tolerated residue in naturally occurring Brevinin-1 heptapeptide rings. In heptapeptide ring containing Brevinin-1 type peptides, the residue at P5 was preferentially lysine or arginine. The most prevalent P2 and P6 residue were also the lysine. Therefore, for most Brevinin-1 family peptides, the “Rana-Box” provides at least two net charges. They may play a significant role in the electrostatic interactions between cationic peptide and anionic cancer or bacterial membranes. The specific membrane binding model of Brevinin-1 family peptides may be the same as the previous model in which the helical kink leads to a diagonal binding of the N-terminal hydrophobic residues in the concave of the helix deeply combined with the membrane core and cationic clusters of “Rana-Box” in the convex of the helix being exposed on the membrane surface [26].

## Conclusion

Brevinin-1GHd is a novel Brevinin-1 family peptide isolated from the skin secretion of *Hylarana guentheri*. Brevinin-1GHd exhibited potent bactericidal activity toward common and drug-resistant bacteria strains and significant anti-proliferation activity against a range of human cancer cells. Furthermore, Brevinin-1GHd displayed rapid bacterial-killing kinetics and considerable capacity in eradicating *S. aureus* and MRSA biofilms. It showed negligible cytotoxicity toward horse red blood cells at MIC concentrations. Brevinin-1GHd also provided a valuable template to further study the relationship of structural parameters and hemolysis of Brevinin-1 type peptides and clues for design of potential alternative antibiotics.

## Abbreviations

AMPs: antimicrobial peptides
CD: circular dichroism
CFU: colony forming unit
LPS: lipopolysaccharide
MALDI-TOF: matrix-assisted laser desorption ionization time-of-flight
MBC: minimum bactericidal concentration
MBEC: minimum biofilm eradication concentration
MBIC: minimum biofilm inhibitory concentration
MHA: Mueller Hinton agar
MHB: Mueller-Hinton broth
MIC: minimal inhibitory concentration
MRSA: methicillin-resistant *Staphylococcus aureus*
NH_4_AC: ammonium acetate
OD: optical density
PBS: phosphate-buffered saline
RACE: rapid amplification of cDNA ends
RP-HPLC: reverse-phase HPLC
TFA: trifluoroacetic acid
TFE: trifluoroehanol
